# Consistency for the tree bootstrap in respondent-driven sampling

**DOI:** 10.1093/biomet/asz067

**Published:** 2020-01-24

**Authors:** A K B Green, T H McCormick, A E Raftery

**Affiliations:** 1 Scottish Government, Atlantic Quay, 150 Broomielaw, Glasgow G2 8LU, UK; 2 Department of Statistics, University of Washington, Seattle, Washington 98195-4322, USA

**Keywords:** Block bootstrap, Consistency, Respondent-driven sampling, Tree bootstrap

## Abstract

Respondent-driven sampling is an approach for estimating features of populations that are difficult to access using standard survey tools, e.g., the fraction of injection drug users who are HIV positive. Baraff et al. (2016) introduced an approach to estimating uncertainty in population proportion estimates from respondent-driven sampling using the tree bootstrap method. In this paper we establish the consistency of this tree bootstrap approach in the case of $m$-trees.

## 1. Introduction

In public health and epidemiology, many populations of interest fall outside the frame of traditional sampling methods. In HIV/AIDS research, for example, studies often focus on injection drug users, female sex workers and men who have sex with men. Members of these groups are considered most at risk for contracting the disease (UNAIDS, 2010). Understanding the rate of infection among these individuals is therefore critical for understanding both the current impact of the disease and for predicting future disease dynamics. Reaching members of such groups with a typical survey instrument, however, is extremely challenging. Individuals may be reluctant to reveal their membership to unfamiliar survey or public health officials, particularly in situations where there is the possibility of prosecution. Further, even if a member were to reveal their status, a simple probability sample from the population would be inefficient. Members of these groups are relatively rare, requiring a large sample size to include members of the group, and an even larger sample to include a subset of members who also know they are HIV positive.

Respondent-driven sampling was proposed by Heckathorn (1997) and is a commonly used method to obtain samples from hard-to-reach groups. Rather than reaching individuals by selecting with some probability from a sampling frame, respondent-driven sampling uses respondents’ networks to recruit members of the hard-to-reach group. Typically, respondent-driven sampling begins with a convenience sample of individuals, known as seeds. These could be, for example, individuals who already have contact with local public health officials. Each seed individual then recruits a preset number of individuals from their network, typically by passing a coupon with a unique identification number. When individuals return to the survey site with a coupon, two things happen. First, individuals are asked about their status, such as whether they are HIV positive. Second, they are themselves instructed to recruit more individuals in the group of interest.

Performing inference using this network-dependent sampling scheme has been a persistent challenge. Salganik & Heckathorn (2004) and Volz & Heckathorn (2008) provided early estimators which rely on the assumption that a respondent’s probability of being included in the sample is proportional to his or her degree. Goel & Salganik (2009) proposed a systematic statistical framework derived by viewing respondent-driven sampling as Markov chain Monte Carlo. Under assumptions, they showed that dependence on the seeds and the network dependence in the sampling decreases in subsequent sampling waves. However, empirical evaluations showed that these methods were not robust to violations of the assumptions required by respondent-driven sampling methods (Gile & Handcock, 2010; Goel & Salganik, 2010). In response, Gile & Handcock (2015) proposed a model-assisted estimator, using realizations of a network model to fill in pieces of the network not captured by respondent-driven sampling. Crawford (2016), meanwhile, focused on properties of the recruitment process, building a probability distribution on the recruitment-induced subgraph.

Two additional recent developments in the respondent-driven sampling literature are particularly relevant for this paper. In both cases, the key realization comes from viewing respondent-driven sampling as a tree, where the nodes in the tree are individuals and links are referrals. First, using this set-up, Baraff et al. (2016) proposed a new variance estimator for respondent-driven samples based on the tree bootstrap. Baraff et al. (2016) tested the new estimator using simulations and found that the method has better performance than existing methods in terms of confidence interval coverage. Next, Rohe (2019) explored the theoretical properties of the respondent-driven sampling tree, in particular by examining the role of design effects, or the additional variation introduced by the sampling strategy. Rohe (2019) concluded that, under assumptions, network sampling processes have a critical threshold based on the rate of referrals and the clustering structure in the network. For cases below the threshold, the design effect is bounded. Past the threshold, however, the design effect grows with the sample size, resulting in slower convergence of the standard error estimator.

## 2. Preliminaries

### 2.1. The respondent-driven sampling process

In an respondent-driven sample, we begin with an initial set of seeds, which can be thought of as root vertices of the recruitment trees. The seeds can be obtained by convenience sampling, since random or other probability sampling is usually not achievable due to the often sensitive nature of the populations in question. In practice, seeds are recruited into the study based on their pre-existing ties with researchers; they are in a sense the visible members of the population. When respondent-driven sampling is performed, often several seeds are initialized so that the recruitment tree is less dependent on a particular choice of seed. This also discourages the sampling procedure from concentrating in a specific community of the network underlying the respondent-driven sampling process. The seeds are provided with recruitment coupons and given the task of recruiting their contacts in the hidden population, with financial incentives for both the recruiter and recruitee. Usually participants are given up to three coupons, to encourage more waves of recruitment, rather than fewer bushier branches, whilst aiming to ensure that the recruitment process continues even if some recruitees do not recruit others. The next wave returns with coupons and they are, in turn, given the task of recruiting the following wave, continuing until no recruitee recruits another or the desired sample size is reached.

### 2.2. The tree bootstrap method for respondent-driven samples

The tree bootstrap method was developed by Baraff et al. (2016) to estimate the sampling distribution of respondent-driven sampling estimators via a resampling approach that requires no extra assumptions on the respondent-driven sampling process. In comparison with standard bootstrap methods, the tree bootstrap method is developed in such a way as to preserve any homophily, or the tendency for similar individuals to be connected, which is generally seen in respondent-driven samples (Heckathorn, 1997). The first step of the method is to resample with replacement from the seeds to create bootstrap sample seeds. For each of the bootstrap sample seeds, we then resample with replacement from their recruits in the original sample. We continue in this manner until all layers of the original sample are exhausted. As noted by Baraff et al. (2016), the tree bootstrap method is similar to bootstrap methods for time series data. To obtain our results we use a framework developed by Kunsch (1989) for the moving block bootstrap, by establishing a similar block structure, allowing us to prove an analogous result to Theorem 3.5 of Kunsch (1989).

We use the term nodes or vertices to refer to individuals or actors, and edges to denote the connections or links between nodes. Following the notation of Rohe (2019), let $\mu_0$ be the true population mean, so that $\mu_0 = {1}/{N} \sum_{i \in G} x(i)$, where $N$ is the population size, $G = \{V(G), E(G)\}$ is the population graph with the population members as vertices and edges corresponding to whether two members are connected such that one could refer the other in the respondent-driven sampling chain or not. We consider all edges as undirected, so that edges are symmetric between individuals. We denote by $x(i)$ the indicator function of population member $i$ of the binary trait, e.g., $x(i)$ is the indicator for whether person $i$ is HIV positive and, following Rohe (2019), we write $i \in G$ synonymously with $i \in V(G)$.

We denote the recruitment forest by $\mathbb{T}$. The nodes of $\mathbb{T}$ are fixed, and will index the variables in our Markov process. For $r,s \in \mathbb{T}$, we have that $ (r,s) $ belongs to the edge set of $\mathbb{T}$ if random population member $W_r \in G$ refers $W_s \in G$. Under a series of assumptions on the underlying population graph, notably connectedness, reciprocity of all ties, and that individuals are recruited with probability proportional to degree, the process is Markov with transition matrix $ P \in [0,1]^{N \times N}$. Rohe (2019) noted that the process behaves as a $(\mathbb{T}, P)$-walk on the population graph, a notion introduced by Benjamini & Peres (1994). This model allows for with-replacement design and, in particular, an individual can recruit the same contact into the study multiple times in the same wave. We observe $X_s = x(W_s)$ for each $s \in \mathbb{T}$.

This Markov process indexed by $\mathbb{T}$ is a collection $\left\{ W_s : s \in \mathbb{T}\right\}$ such that $\mathbb{P}(W_t \mid W_s, W_r )= \mathbb{P}(W_t \mid W_s)$, where $W_r$ is not a descendant of $t$. It is assumed that the walk begins at stationarity. We observe $n$ random members of the population, where repeats are possible, it is possible that $W_r=W_s, r =\hspace{-.8em}|\ s \in \mathbb{T}$, and the corresponding random variables are indexed by the nodes of the recruitment tree.

### 2.3. A transformation between the sample mean and respondent-driven sampling estimators

A simple transformation is possible between the sample mean and several estimators that can be used with respondent-driven sampling Rohe (2019). We will explain this relationship below, but for full details of this set-up, refer to Rohe (2019). We outline this transformation here and will use it in the subsequent section where we show our main result in terms of the sample estimator.

Consider first the sample mean set-up. The sample mean of observations is

$$\begin{equation*}
    \bar{X}_n = \frac{1}{n} \sum_{s \in \mathbb{T}}X_s.
\end{equation*}$$

This is an unbiased estimator of $\mu$, where

$$\begin{equation*}
    \mu = \sum_{i \in G} x(i) \pi_i
\end{equation*}$$

and $\pi_i$ is the sampling probability of person $i$.

We now move to the set-up for respondent-driven sampling. We want to estimate $\mu_0$ where

$$\begin{equation*}
    \mu_0 = \frac{1}{N}\sum_{i \in G} x(i).
\end{equation*}$$

If $\pi_i$ is the same for all $i$, then $\mu = \mu_0$. An unbiased estimator of $\mu_0$ is the inverse probability weighted estimator, a Horvitz–Thompson estimator. The issue is that it requires computation of $\sum_{j=1}^N {\rm deg}_G(j)$, where ${\rm deg}_G(j)$ is the degree, or network size, of person $j$. This quantity is generally unknown. The inverse probability weighted estimator is

$$\begin{equation*}
\hat{\mu}_{\rm IPW} = \frac{\sum_{j=1}^N {\rm deg}_G(j)}{nN}\sum_{s \in \mathbb{T}}\frac{X_s}{{\rm deg}_G(W_s)} .
\end{equation*}$$

The Volz–Heckathorn estimator (Volz & Heckathorn, 2008) is an approximation to the inverse probability weighted estimator, and is a ratio of Horvitz–Thompson estimators given by $\hat{\mu}_{\rm VH}$, where

$$\begin{equation*}
    \hat{\mu}_{\rm VH} = \frac{1}{nH} \sum_{s \in \mathbb{T}} \frac{X_s}{{\rm deg}_G(X_s)},
\end{equation*}$$

where $H$ is defined by

$$\begin{equation*}
\frac{1}{n} \sum_{s \in \mathbb{T}} \frac{1}{{\rm deg}_G(X_s)} 
\end{equation*}$$

and ${\rm deg}_G(X_s)$ is the number of contacts $X_s$ has in the population. As noted by Rohe (2019), the theorem below which studies $\bar{X}_n$ can be applied to $\hat{\mu}_{\rm IPW}$ by defining $x^\pi(i) = {x(i)}/{\pi_iN}$, since the sample mean of the variables $X_s^\pi$ indexed by the tree is precisely the IPW estimator.

## 3. Consistency of the tree bootstrap

### 3.1. Notation and main theorem

We consider the tree bootstrap under the $m$-tree assumption. We consider the case where for sample size $n$ there are $k=k(n)$ seeds, and each of these seeds gives rise to a seed-rooted recruitment tree which is a complete $m$-ary tree of height $h=h(n)$. Note that $n = k \ell$, where $\ell = {m^{h+1}-1}/{m-1}$. We introduce the following notation. Let $X_s$ be the value of the binary trait of interest indexed by $s \in \mathbb{T} = \cup_{j=1}^k \mathbb{T}_j$. $\mathbb{T}$ has $n$ vertices; we suppress this from the notation throughout. These quantities are random in the respondent-driven sampling process, but when analysing the bootstrap we condition on the original sample so that these are treated as fixed. Let $\mu=\sum_{i \in G} x(i) \pi_i$. Let $\bar{X}_n$ be the sample mean of a sample $X_1, \dots, X_n$. Let $\mathbb{E}_*(\bar{X}_n^*)$ be the expectation of the average from the tree bootstrap method; $\mathbb{E}(\bar{X}_n)$ is the expectation of the average from the respondent driven sample. We have that ${\rm var}_{\rm RDS}$ is the variance under respondent-driven sampling and ${\rm var}_*$ is the variance under the tree bootstrap method.

The Volz–Heckathorn estimator of $\mu$ is the corresponding Hájek estimator of the inverse probability weighted estimator (Rohe, 2019). If the degrees of the sampled individuals are fixed and known, the inverse probability weighted estimator is indeed a simple function of the reweighted sample mean. We will thus consider results for the sample mean and refer the reader to § 2 for a simple transformation of this estimator, to which our result can be applied and follows immediately by use of the delta method for the bootstrap (van der Vaart, 2000, Theorem 23.5). In other words, the consistency is invariant to the inverse probability weighted transformation. Our result thus holds for the inverse probability weighted estimator, provided the degrees of the sampled individuals are fixed and known, and, as noted in § 2.3, the inverse probability weighted estimator is the Volz–Heckathorn estimator if the normalizing constant ${1}/{N} \sum_{j=1}^N {\rm deg}_G(j)$ is known. Remarks are made at the end of the proof as to how relaxing this assumption alters the conditions necessary for consistency to hold, but at this stage we require knowledge of the normalizing constant to use the properties of the transformation.

The goal is to show consistency of the tree bootstrap method under assumptions on the respondent-driven sampling process and the bootstrap method. That is, under such assumptions the asymptotic distribution of the tree bootstrap estimator is the same as that of the original estimator. We will show the following:

Theorem 1. *Suppose the following conditions hold:*

(i) *$n^{-1/2} \sum_{s\in \mathbb{T}} \left(X_s-\mu\right) \stackrel{d}{\to} N(0, \rho^2)$ for some $\rho^2 \in \mathbb{R}^{>0}$, i.e., a central limit theorem holds for the respondent-driven sampling tree-structured data. For this to hold, we must be under the critical threshold;*
(ii) $n \hat{\sigma}_{\infty,n}^2 \to \rho^2$ almost surely, where $\hat{\sigma}_{\infty,n}^2:=\lim_{B \to \infty} \hat{\sigma}^2_{B,n}={\rm var}_{*}(\bar{X}_n^*)$ is the exact bootstrap variance estimator of the mean, conditional on the sample $X_1, \dots, X_n$;
(iii) *$\ell(n)=o(\surd{n})$ and $\ell(n) \to \infty$.*

*Under the conditions above,*

$$\begin{equation*}
    \sup_{x \in \mathbb{R}} \big| \mathbb{P}\left(\bar{X}_n^*-\bar{X}_n\leqslant x \mid X_1, \dots, X_n \right)-\mathbb{P}\left(\bar{X}_n- \mu \leqslant x\right)\big| \to 0 
\end{equation*}$$

*almost surely.*

Remark 1. This result gives uniform convergence to 0 of the absolute difference between the distribution of $\bar{X}_n - \mu$ and its bootstrap approximation. This establishes bootstrap consistency in the sense that the bootstrap estimator of the distribution of the sample mean is strongly consistent with respect to the Kolmogorov metric. In turn, this establishes consistency of bootstrap confidence intervals.

Remark 2. A consequence of the $m$-tree assumption is that, unlike in the typical tree bootstrap case, the number of sampled vertices does not change between bootstrap samples.

Remark 3. Condition 1 of Theorem 1 requires a central limit theorem result for the respondent-driven sampling tree process. Li & Rohe (2017) gave conditions for such a limit theorem for 2-trees and postulated conditions for the general $m$-tree case. As an example, Li & Rohe (2017) derived the conditions under which the 2-tree respondent-driven sampling process taken from a stochastic block model (Lorrain & White, 1971; Holland et al., 1983) and outcomes that are symmetric and related to block membership satisfy such a limit theorem. Say that the probability of a connection between two nodes in different blocks is $r$, and the probability of connection between two nodes in the same block is $p$. Then, in the case of a model where there are $2K$ blocks, and each block contains $N/(2K)$ vertices, Li & Rohe (2017) showed that such a limit theorem holds if

$$\frac{1}{2K}<\tilde{p}<\frac{1}{2K}+\frac{1}{2\surd{2}}, $$

where $\tilde{p}=p/\{p + r(K-1)\}$. Yan et al. (2019) explored limit behaviour of estimators when the respondent-driven sampling process is above the critical threshold. We leave this as an area for future work.

Before turning to the proof of our main result, we introduce some more notation. The recruitment forest consists of $k$ subtrees, rooted at the seeds. Let $\mathbb{T}_j$ denote the recruitment subtree corresponding to the $j$th seed. Due to *Theorem* 1 (iii), $k$ is a function of $n$ and the recruitment forest is also of $n$ vertices, but we suppress this in our notation. Let $K$ be the total number of possible resampled trees across all seeds, where we distinguish orders of children. The quantity $K$ is a multiple of $k$, so that $K = ak$, where $a \in \mathbb{N}$. Enumerating the $a$ possible resampled trees of $\mathbb{T}_j$, let $\mathbb{T}_{j,p}$ be the $p$th possible resampled tree from $\mathbb{T}_j$, for $p=1, \dots, a$. The quantity $\mathbb{T}_{j,p}$ is an $\ell$-tuple, subject to particular constraints, of vertices in $\mathbb{T}_j$.

### 3.2. Proof of Theorem 1

We need to show that $\surd{n}(\bar{X}_n^* - \bar{X}_n) \stackrel{d}{\to} N(0, \rho^2)$, almost surely. Our argument proceeds as follows. First, by simply adding and subtracting $\mathbb{E}_*[\bar{X}_n^*]$, the expectation of the bootstrap mean, we can write

$$
\surd{n}(\bar{X}_n^* - \bar{X}_n)=\surd{n}\{\bar{X}_n^* - \mathbb{E}_*(\bar{X}_n^*)\}-\surd{n}\{\mathbb{E}_*(\bar{X}_n^*)- \bar{X}_n\}.
$$

Using the above expression, we first deal with the term involving only the bootstrap and show that $\surd{n}\{\bar{X}_n^* - \mathbb{E}_*(\bar{X}_n^*)\}$ converges to a normal limiting distribution with mean zero and variance $\rho^2$. We do this with a Lindeberg–Feller central limit theorem, where the main condition is given in (1) below. In the process, we argue that the the bootstrap sample average is unbiased for the respondent-driven sample average in Proposition 1. Next, we show that the respondent-driven sample average converges to the true population average in Lemma 2. These latter two results combine to yield that the rightmost term in the expression above, the difference between the expected bootstrap average and the respondent-driven sampling average, goes to zero. The expression is, therefore, the combination of a term that converges in distribution to a limiting normal distribution, $\surd{n}\{\bar{X}_n^* - \mathbb{E}_*(\bar{X}_n^*)\}$, and one that goes almost surely to zero, $\mathbb{E}_*(\bar{X}_n^*)-\surd{n}\{\mathbb{E}_*(\bar{X}_n^*) - \bar{X}_n\}$. By Slutsky’s theorem we have the result; see the Supplementary Material.

We now show the details of our argument. As described above, the first portion of the proof deals with $\surd{n}\{\bar{X}_n^* - \mathbb{E}_*(\bar{X}_n^*)\}$. We borrow the following construction from the framework used by Kunsch (1989) for the moving block bootstrap and define $C_n := \surd{n}\{\bar{X}_n^* - \mathbb{E}_*(\bar{X}_n^*)\}$. As outlined in the previous paragraph, the first step is to prove that $C_n \stackrel{d}{\to} N(0, \rho^2)$. To show this, we first observe that

$$\begin{equation*}
\bar{X}_n^*=\frac{1}{k}\sum_{i=1}^k U_{n,i},
\end{equation*}$$

where $U_{n,i}$ are independent and identically distributed random variables with distribution

$$\begin{equation*}
     \mathbb{P}\bigg(U_{n,i} = \frac{\sum_{s \in \mathbb{T}_{j,p}} X_{s}}{\ell}\bigg)=  \mathbb{P}\bigg(U_{n,i} = \frac{\sum_{s \in \mathbb{T}_{j,p}} X_{s}}{n/k}\bigg) = \frac{1}{K} 
 \end{equation*}$$

for $j \in \left\{1, \dots, k\right\}$, $ p \in \left\{1, \dots, a\right\}$, where $\mathbb{T}_{j,p}$ is the $p$th possible resampled subtree from $\mathbb{T}_j$ and $s \in \mathbb{T}_{j,p}$. Now, since $n \hat{\sigma}^2_{\infty,n} \to \rho^2$ almost surely, ${\rm var}_*(C_n) \stackrel{\text{a.s.}}{\to} \rho^2$ also. We now show that we can apply the Lindeberg–Feller central limit theorem, to the collection of independent random variables whose sum is equal in distribution to $C_n$. Note that $\mathbb{E}_*(\bar{X}_n^*) = \mathbb{E}(U_{n,i})$, so that

$$\begin{align*}
 C_n  = \surd{n}\{\bar{X}_n^* - \mathbb{E}_*(\bar{X}_n^*)\}= \frac{\ell k}{\surd{n}}\left\{\frac{1}{k} \sum_{i=1}^k U_{n,i} - \mathbb{E}_*(\bar{X}_n^*)\right\}
 =\frac{\ell}{\surd{n}} \sum_{i=1}^k \{U_{n,i} - \mathbb{E}(U_{n,i})\}.
\end{align*}$$

We thus aim to show that for all $\epsilon >0$,

(1) \begin{align*}\label{kunsch linde} \sum_{i=1}^k \mathbb{E}\{Z_{n,i}^2 \mathbb{I} \left(|Z_{n,i}| > \epsilon \right) \} \to 0 \end{align*}

as $n \to \infty$, where $Z_{n,i} = \frac{1}{\surd{n}}\ell\{U_{n,i}- \mathbb{E}(U_{n,i})\}$, noting that since $n=k\ell$ and $ \ell=o(\surd{n})$ then as $n \to \infty, k(n) \to \infty$ also.

To establish the Lindeberg–Feller condition, we will first argue that the expectation is bounded, and then that the probability that the indicator is nonzero vanishes as $n$ goes to infinity. To begin, let $\epsilon >0$. Since the $Z_{n,i}$ are independent and identically distributed, it suffices to show $\mathbb{E}\{Z_{n,1}^2 \mathbb{I}\left(|Z_{n,1}|> \epsilon\right)\}=o(k^{-1})$. Since ${\rm var}(Z_{n,1}) < \infty$ and $\mathbb{E}(Z_{n,1})=0$, it follows that $\mathbb{E}(Z_{n,1}^2)<\infty$. Moreover, $\mathbb{E}(Z_{n,1}^2)={\rm var}_*(C_n)/k$, so by assumption $k \mathbb{E}(Z_{n,1}^2)$ is still finite, since

$$\begin{align*}
n {\rm var}_*(\bar{X}_n^*) \stackrel{\text{as}}{\to} \rho^2
&\implies {\rm var}_*(C_n) \stackrel{\text{as}}{\to} \rho^2.
\end{align*}$$

We now move to the second part of the argument, which consists of the following lemma.

Lemma 1. *We have that* $\lim_{n \to \infty} \mathbb{P}(|Z_{n,\,1}| > \epsilon) =0. $

*Proof.* Consider

$$\begin{align*}
    \mathbb{P}(|Z_{n,1}| > \epsilon) & = \mathbb{P}\left[\bigg|\frac{\ell}{\surd{n}}\{U_{n,1}-\mathbb{E}(U_{n,1})\}\bigg| > \epsilon \right]
    = \mathbb{P}\left\{\bigg|\ell U_{n,1} -\ell  \mathbb{E}(U_{n,1})\bigg| > \epsilon \surd{n}\right \}\\
    &=  \frac{1}{K} \# \left\{ (j,p) \in [a] \times [k] : \bigg|\sum_{s \in \mathbb{T}_{j,p}} X_s - \ell \mathbb{E}_*(\bar{X}_n^*) \bigg| > \epsilon \surd{n} \right\}\\
    & \leqslant \mathbb{I}\left\{\max_{(j,p) \in [k] \times [a]} \bigg|\sum_{s \in \mathbb{T}_{j,p}} X_s - \ell \mathbb{E}_*(\bar{X}_n^*) \bigg| > \epsilon \surd{n} \right\}\!.
\end{align*}$$

Note that

$$\begin{align*}
    \max_{(j,p) \in [k] \times [a]} \bigg|\sum_{s \in \mathbb{T}_{j,p}} X_s - \ell \mu \bigg| & = \max_{(j,p) \in [k] \times [a]} \bigg|\sum_{s \in \mathbb{T}_{j,p}} \left(X_s - \mu\right) \bigg|\\
    & \leqslant \max_{(j,p) \in [k] \times [a]} \sum_{s \in \mathbb{T}_{j,p}} \big| X_s -  \mu \big|
    \leqslant |\mathbb{T}_{j,p}| = \ell.
\end{align*}$$

Moreover, we have

$$\begin{align*}
    \bigg|\sum_{s \in \mathbb{T}_{j,p}} X_s - \ell \mathbb{E}_*(\bar{X}_n^*) \bigg| & = \bigg|\sum_{s \in \mathbb{T}_{j,p}} X_s - \ell \bar{X}_n + \ell \bar{X}_n - \ell \mathbb{E}_*(\bar{X}_n^*) \bigg| \\
    & \leqslant \bigg|\sum_{s \in \mathbb{T}_{j,p}} X_s - \ell \bar{X}_n \bigg| + \bigg|\ell \bar{X}_n - \ell \mathbb{E}_*(\bar{X}_n^*) \bigg|.
\end{align*}$$

We next introduce a proposition that will complete the Lindeberg–Feller argument.

Proposition 1. For any dataset $X_1, \dots, X_n$, $\bar{X}_n^*$ is unbiased for $\bar{X}_n$ under the tree bootstrap.

The proof of Proposition 1 requires defining several items from graph theory, and is given in the Supplementary Material. It follows that

$$\begin{equation*}
    \frac{\ell}{\surd{n}}|\mathbb{E}_*(\bar{X}_n^*)- \bar{X}_n| \to 0
\end{equation*}$$

almost surely by Proposition 1. Now, by assumption, $\ell = \frac{m^{h+1}-1}{m-1} =o(\surd{n})$. With that, the proof of Lemma 1 is complete.$\square$

The proof of the Lindeberg–Feller condition (1) is complete. All that remains to apply Slutsky’s theorem and establish Theorem 1 is to show that $\bar{X}_n$ converges to the true population mean, $\mu$, under the conditions we have set out. The proof of Theorem 1, therefore, concludes with the following lemma.

Lemma 2. *We have that* $\bar{X}_n \to \mu$*almost surely*.

The proof of Lemma 2 is given in the Supplementary Material. With Lemma 2 we also have the proof of Theorem 1.

Remark 4. If we assume that ${1}/{N} \sum_{j=1}^N {\rm deg}_G(j)$ is known, the proof of Lemma 2 extends to the Volz–Heckathorn estimator easily as we still have unbiasedness. If this quantity is unknown, the Volz–Heckathorn estimator is the Hájek estimator (Rohe, 2019), which is asymptotically unbiased under the Markov assumptions made in the respondent-driven sampling literature. Then, in the proof of Lemma 2, $c_n = \mathcal{O} \left(\frac{1}{n^2}\right) + \frac{1}{n}\{\mu-\mathbb{E}(\bar{X}_n)\}^2$. By asymptotic unbiasedness, we certainly have that $\{\mu-\mathbb{E}(\bar{X}_n)\}^2=o(1)$, but we need to establish a faster rate of convergence. In particular, we require

$$\begin{align*} \label{hajek}
    \{\mu - \mathbb{E}(\bar{X}_n)\}^2 \to 0
\end{align*}$$

at a rate such that $\sum_{n=1}^\infty \frac{1}{n} \{\mu - \mathbb{E}(\bar{X}_n)\}^2$ converges. A sufficient condition is that for any $\epsilon >0$, $\{\mu - \mathbb{E}(\bar{X}_n)\}^2 = \mathcal{O}\left(\frac{1}{n^\epsilon}\right)$, where $ f(n)= \mathcal{O}\{g(n)\}$ is defined in the standard way: $ \exists M >0$ such that $ f(n)\leqslant Mg(n) \, \forall n$. Under this condition, the consistency result here implies that an application of Slutsky’s theorem, given as Theorem S1 in the Supplementary Material, extends Theorem 1 to the Volz–Heckathorn estimator as well.

## Supplementary Material

asz067_Supplementary_Data

## Supplementary material

Supplementary material available at *Biometrika* online contains proofs of the main results and some auxiliary theorems.
