# Supplementary material for: Consistency for the tree bootstrap in respondent-driven sampling
Source: Biometrika. 2020 Jan 24;107(2):497–504. doi: 10.1093/biomet/asz067 (PMC7228542; doi:10.1093/biomet/asz067)
Supplement: asz067_Supplementary_Data [file asz067_supplementary_data.pdf]

# Supplementary Material for "Consistency for the tree bootstrap in respondent-driven sampling"

A. Green, T. H. McCormick and A. E. Raftery

# 1 Proofs

**Proof of Lemma 2:** We will use Lyon's result (Lyons, 1988) for bounded random variables (see Theorem 4). Consider $V_s = X_s - \mu$. The first two conditions of Theorem 4 hold for $V_s$ since the $X_s$ are binary, and $\mu \in [0,1]$. It remains to check that $\sum_{n=1}^{\infty} c_n < \infty$, where $c_n = \frac{1}{n}\mathbb{E}\left(\frac{1}{n}\sum_{s\in\mathbb{T}} V_s\right)^2$. Consider

$$\begin{aligned}
nc_n &= \mathbb{E}(\bar{X}_n - \mu)^2 \\
&= \mathbb{E}(\bar{X}_n - \mathbb{E}\bar{X}_n + \mathbb{E}\bar{X}_n - \mu)^2 \\
&= \mathbb{E}(\bar{X}_n - \mathbb{E}\bar{X}_n - (\mu - \mathbb{E}\bar{X}_n))^2 \\
&= \mathbb{E}(\bar{X}_n - \mathbb{E}\bar{X}_n)^2 - 2(\mu - \mathbb{E}\bar{X}_n)\mathbb{E}[\bar{X}_n - \mathbb{E}\bar{X}_n] + \mathbb{E}(\mu - \mathbb{E}\bar{X}_n)^2 \\
&= \mathrm{Var}_{RDS}(\bar{X}_n) + (\mu - \mathbb{E}\bar{X}_n)^2.
\end{aligned}$$

We thus have $c_n = \frac{1}{n}\mathrm{Var}_{RDS}(\bar{X}_n)$ since the estimator $\bar{X}_n$ for $\mu$ is unbiased, as we assume that the seeds are sampled from the stationary distribution (as is the case extensively throughout the literature). So in this case the result is immediate. The first term on the RHS is precisely the variance given in Theorem 2.1 of Rohe (2019). Under the critical threshold, Rohe (2019) shows that for $m$-trees

$$\mathrm{Var}_{RDS}(\bar{X}_n) = \mathcal{O}\{\mathrm{Var}_{\pi}(\bar{X}_n)\} = \mathrm{Var}\left\{\frac{1}{n}\sum_{i=1}^{n} x(W_i)\right\}, \tag{1}$$

where $W_i$ are independent random samples from distribution $\mathbb{P}(W_i = j) = \pi_j$ given by the stationary distribution. Thus,

$$\mathrm{Var}_{RDS}(\bar{X}_n) = \frac{1}{n}\mathrm{Var}\{x(W)\}, \tag{2}$$

where $W$ has this distribution. Since $x(W)$ is binary, we can apply Popoviciu's Inequality for variances which states that a random variable taking values on $[m, M]$ has variance bounded by $\frac{(M-m)^2}{4}$ [or just note $v = p(1-p)$ is maximised at $p = 1/2$]. Thus completes the proof of Lemma 2.

**Proof of Proposition 1:** We have the following expression for $Y_n := \sqrt{n}\{\mathbb{E}_*(\bar{X}_n^*) - \bar{X}_n\}$. It then follows that

$$Y_n = \sqrt{n}\{\mathbb{E}_*(\bar{X}_n^*) - \bar{X}_n\} \tag{3}$$

$$= \sqrt{n}\{\mathbb{E}(U_{n1}) - \bar{X}_n\} \tag{4}$$

$$= \sqrt{n}\left(\frac{k}{Kn}\sum_{j=1}^{k}\sum_{p=1}^{a}\sum_{s\in\mathbb{T}_{j,p}} X_s - \frac{1}{n}\sum_{j=1}^{k}\sum_{s\in\mathbb{T}_j} X_s\right) \tag{5}$$

$$= \sqrt{n}\left(\frac{1}{an}\sum_{j=1}^{k}\sum_{p=1}^{a}\sum_{s\in\mathbb{T}_{j,p}} X_s - \frac{1}{n}\sum_{j=1}^{k}\sum_{s\in\mathbb{T}_j} X_s\right) \tag{6}$$

$$= \frac{1}{\sqrt{n}}\sum_{j=1}^{k}\left(\frac{1}{a}\sum_{p=1}^{a}\sum_{s\in\mathbb{T}_{j,p}} X_s - \sum_{s\in\mathbb{T}_j} X_s\right). \tag{7}$$

We note from the above that in proving the Proposition it is sufficient to prove the following Lemma.

**Lemma 1** *Conditional on the height of the recruitment trees, $h$, each vertex $s \in \mathbb{T}_j$ appears exactly $a = a(h)$ times across all of the $a$ resampled subtrees $\mathbb{T}_{j,1}, \ldots, \mathbb{T}_{j,a}$.*

Before proving the Lemma, we introduce some graph theoretical concepts we will use:

**Definition 1 (*uv*-path)** *For two vertices $u, v$ of a graph $G$, a $uv$-path is an alternating vertex-edge sequence $v_0 e_0 v_1 e_1 \ldots v_i$ with endpoints $v_0 = u$ and $v_i = v$ such that each of the vertices are distinct from one another.*

**Definition 2 (Length of a *uv*-path)** *The length of a $uv$-path in a graph $G$ is defined as the number of edges in the path.*

**Definition 3 (Graph distance)** *The graph distance between two vertices $u, v \in G$ is defined as the length of a shortest $uv$-path in $G$.*

**Definition 4 (Subtree rooted at $u$)** *We define a subtree rooted at $u$ as follows. Consider the case where we are mid-construction of a resampled recruitment tree, and we have just resampled $u$ in our realization. At this step we then consider vertex $u$ as a secondary root; the root of the subtree subtended from vertex $u$. All succeeding vertices added to the tree by an edge either to vertex $u$ or descendants of vertex $u$ form this induced subtree, known as the subtree rooted at $u$.*

**Remark 1** *If $G$ is a tree, then any vertices $u, v \in G$ have a unique path connecting them, and so the graph distance between $u$ and $v$ is the length of the unique $uv$-path in $G$.*

**Proof of Lemma 1** Let $O_j$ denote the $j^{th}$ seed for the purpose of this proof. The graph distance between $s \in \mathbb{T}_j$ and $O_j$ is preserved upon resampling, i.e. if $s \in \mathbb{T}_{j,p}$ then $d_{\mathbb{T}_{j,p}}(s, O_j) = d_{\mathbb{T}_j}(s, O_j)$. Note also that we distinguish between resampled trees with differing left-right ordering of children. The proof is a simple induction on the distance between $s$ and $O_j$, $d_{\mathbb{T}_j}(s, O_j)$. We can assume without loss of generality that $h \geq 1$, else $a = 1$. Consider first the trivial base case: $d_{\mathbb{T}_j}(s, O_j) = 0 \iff s = O_j$. There are $a$ enumerations of resampled subtrees rooted at $O_j$, and $O_j$ appears exactly once in each of them. Assume the inductive hypothesis: if $d_{\mathbb{T}_j}(s, O_j) = k - 1 < h$ then $s$ appears exactly $a$ times across all possible subtrees rooted at the $j^{th}$ seed. Suppose $s'$ is such that $d_{\mathbb{T}_j}(s', O_j) = k \leq h$. In $\mathbb{T}_j$, $s'$ has exactly one ancestor, say $s$, and it holds that $d_{\mathbb{T}_j}(s, O_j) = k - 1$. Now, $s$ appears exactly $a$ times across all possible resampled subtrees rooted at $O_j$, and has $m$ children in $\mathbb{T}_j$ – one of which is $s'$. Given that we have resampled $s$, we can consider the next step of resampling of the subtree rooted at $s$ as a realization of a binomial random variable with $m$ draws from a population of $m$ children of $m - 1$ failures and 1 success, corresponding to $s'$. The expectation of this random variable is 1. Since we enumerate each realization, it follows from the inductive hypothesis that $s'$ appears exactly $a$ times across all of the $a$ resampled subtrees also.

**Remark 2** *By the Lemma, the summand in Equation (7) is exactly 0.*

# 2 Auxiliary theorems

Here we state several theorems that we use in our proof, but are due to others.

**Theorem 2 (Slutsky 1925 )** *If $A_n \stackrel{d}{\to} A$ and $B_n \stackrel{d}{\to} c$, where c is constant, then $A_n + B_n \stackrel{d}{\to} A + c$.*

**Theorem 3 (Lindeberg 1922 , Feller 1945 )**
*Let $(Z_{ni})_{1 \leq i \leq n}$ be independent for each $n$ and suppose $\mathbb{E}(Z_{ni}) = 0$ and $\mathbb{E}(Z_{ni}^2) < \infty$. Let $T_n := \sum_{i=1}^n Z_{ni}$, $\rho_n^2 = \sum_{i=1}^n \mathrm{Var}(Z_{ni})$ and suppose the following hold:*

1. $\exists \rho^2 \in [0, \infty)$ *such that*
$$\sum_{i=1}^n \mathbb{E}(Z_{ni}^2) \stackrel{n \to \infty}{\to} \rho^2$$

2. *(the Lindeberg condition)* $\forall \epsilon > 0$,
$$\frac{1}{\rho_n^2} \sum_{i=1}^n \mathbb{E}\left(Z_{ni}^2\right) \mathbb{I}\left\{|Z_{ni}| > \epsilon \rho_n\right\} \stackrel{n \to \infty}{\to} 0$$

*Then $T_n \stackrel{d}{\to} N(0, \rho^2)$.*

**Theorem 4 (Lyons 1988 )** *If $\{V_i\}_{i=1}^{\infty}$ is a real-valued sequence of random variables on $(\Omega, \mathbb{P})$ such that*

$$\mathbb{E}V_i^2 = \int_{\Omega} V_i(\omega)^2 d\mathbb{P}(w) \leq 1. \tag{8}$$

*Then, if $|V_i| \leq 1$ almost surely, and*

$$\sum_{n=1}^{\infty} \frac{1}{n} \mathbb{E} \left( \frac{1}{n} \sum_{i=1}^{n} V_i \right)^2 < \infty, \tag{9}$$

*it holds that*

$$\lim_{n \to \infty} \frac{1}{n} \sum_{i=1}^{n} V_i = 0 \tag{10}$$

*almost surely.*

# Bibliography


Feller, W. (1945). The fundamental limit theorems in probability. *Bulletin of the American Mathematical Society*, *51*(11), 800–833.

Lindeberg, J. (1922). Eine neue Herleitung des Exponentialgesetzes in der Wahrscheinlichkeitsrechnung. *Math. Z.*, *15*(1), 211–225.

Lyons, R. (1988). Strong laws of large numbers for weakly correlated random variables. *The Michigan Mathematical Journal*, *35*(3), 353–359.

Rohe, K. (2019). A critical threshold for design effects in network sampling. *The Annals of Statistics*, *47*(1), 556–582.

Slutsky, E. (1925). Über stochastische Asymptoten und Grenzwerte. *Metron*, *5*, 3–89.
